# Divergence and Conservation of the Major UPR Branch IRE1-*bZIP* Signaling Pathway across Eukaryotes

**DOI:** 10.1038/srep27362

**Published:** 2016-06-03

**Authors:** Lingrui Zhang, Changwei Zhang, Aiming Wang

**Affiliations:** 1 London Research and Development Centre, Agriculture and Agri-Food Canada, London, ON, N5V 4T3, Canada

## Abstract

The unfolded protein response (UPR) is crucial to life by regulating the cellular response to the stress in the endoplasmic reticulum (ER) imposed by abiotic and biotic cues such as heat shock and viral infection. The inositol requiring enzyme 1 (IRE1) signaling pathway activated by the IRE1-mediated unconventional splicing of *HAC1* in yeast, *bZIP60* in plants and *XBP1* in metazoans, is the most ancient branch of the UPR. In this study, we systematically examined yeast IRE1p-*HAC1*, plant IRE1A/IRE1B-*bZIP60* and human hIRE1-*XBP1* pairs. We found that, unlike bZIP60, XBP1 is unable to functionally swap HAC1p in yeast, and that the inter-species heterotypic interactions among HAC1p, bZIP60 and XBP1 are not permitted. These data demonstrate evolutionary divergence of the downstream signaling of IRE1-*bZIP*. We also discovered that the dual cytosolic domains of plant IRE1s act *in vivo* in a mechanism consistent with IRE1p and hIRE1, and that plant IRE1B not only interacts with IRE1p but also forms typical IRE1 dynamic foci in yeast. Thus, the upstream components of the IRE1 signaling branch including IRE1 activation and action mechanisms are highly conserved. Taken together these data advance the molecular understanding of evolutionary divergence and conservation of the IRE1 signaling pathway across kingdoms.

Upon translation, newly synthesized proteins are loaded in an unfolded state into the lumen of the endoplasmic reticulum (ER), where they undergo folding and posttranslational modifications aided by ER-resident chaperones to reach maturity^1^,^2^,^3^. The load of client proteins in the ER in excess of its processing capacity primes ER stress and triggers an ER-to-nucleus signaling pathway termed the unfolded proteins response (UPR)^3^,^4^. Of the three classes of membrane-associated sensor transducers known in mammalian cells, the ER localized type I transmembrane protein, inositol requiring enzyme 1 (IRE1) activates the most ancient and conserved UPR branch^5^,^6^.

IRE1 senses the perturbation in the ER folding environment by its N-terminal luminal domain and conveys the ER stress signal across the membrane to the dual cytosolic effectors: linked kinase and RNase domains^7^,^8^,^9^,^10^. The unique output of the IRE1 signaling is the RNase-mediated site-specific cleavage of an mRNA, the product of *HAC1* in yeast^11^, *XBP1* in metazoan^12^,^13^, and *bZIP60* in plants^14^,^15^. The un-spliced mRNA precursors of *HAC1 (HAC1* U) and *XBP1 (XBP1* U) harbor a characteristic hairpin composed of two 7-nt loops, in which a scissile bond 3′ of a guanosine in position 3 of each loop is cleaved by the RNase activity of IRE1^16^. The resulting mRNA halves are joined by the tRNA ligase Trl1 in yeast^17^, or the RTCB tRNA ligase complex in metazoans^18^,^19^,^20^, to produce the spliced form of *HAC1 (HAC1* S) and *XBP1 (XBP1* S), respectively. The encoded HAC1 S and *XBP1* S proteins, both obtaining a transcriptional activation domain (AD) at their C-termini due to the splicing-mediated frame-shift, activate as transcriptional factors the expression of numerous genes to mitigate the ER stress^21^,^22^,^23^.

Like *HAC1* and *XBP1* mRNAs, the un-spliced *bZIP60 (bZIP60* U) mRNA precursor can fold into an IRE1 recognition site composed of two stem loops, each possessing the bases at three positions strictly conserved from yeast to mammalians^14^,^15^,^24^,^25^,^26^. The stem loop structure of *bZIP60* U is capable of protruding the unconventional cleavage sites to the catalytic sites of IRE1, which is well-defined in the IRE1-dependent splicing of *HAC1* and *XBP1* mRNAs^25^,^27^. Upon IRE1A or/and IRE1B activation by ER stress, a 23-bp fragment of *bZIP60* U is spliced out to generate the spliced *bZIP60 (bZIP60* S)^14^,^26^,^28^. The cleaved 5′ and 3′ fragments can be rejoined *in vitro* by the *Arabidopsis* tRNA ligase RLG1^29^. The bZIP60 S protein differs in bZIP60 U by lacking the single transmembrane domain (TMD) and thus becomes an active transcription factor that up-regulates the UPR target genes^14^,^26^,^28^. Apparently, the IRE1-mediated mRNA splicing is a conserved strategy for the IRE1 signaling across eukaryotes^30^.

In yeast, the IRE1p-mediated splicing of *HAC1* U is a stepwise process. Upon ER stress, oligomeric assembly of the ER-luminal domain induces IRE1p clustering in the ER membrane, facilitating the formation of the discrete foci of higher-order oligomers and the docking of *HAC1* U mRNA onto a positively charged motif that is in proximity to the kinase/RNase and transmembrane domains^31^,^32^,^33^,^34^. *HAC1* U mRNA docking also depends on a conserved bipartite element in its 3′ untranslated region (UTR) and is a prerequisite for subsequent processing by the RNase domain of IRE1p^32^. The precisely controlled molecular process leading to the unconventional splicing has been posited to contribute to efficiency and selectivity and, thus, fidelity in UPR signaling^31^,^32^,^34^.

Different from *XBP1* U and *bZIP60* U that are translated, *HAC1* U mRNA cannot be translated due to the base-pairing interaction between the unconventional intron and 5′ UTR^22^,^35^. In mammalian cells, the translation of *XBP1* U under normal conditions originates a hydrophobic patch on the *XBP1* U nascent chain^36^. Due to the translational pausing, the *XBP1* U mRNA-ribosome-nascent chain (R-RNC) complex is temporarily frozen, by which the hydrophobic region of the nascent chain protrudes from the ribosome tunnel to associate with the ER membrane^36^,^37^. This leads to the recruitment of the R-RNC complex and, thus *XBP1* U mRNA, to the vicinity of hIRE1, allowing the XBP1 U mRNA to be efficiently spliced by hIRE1^36^,^37^. Apparently, ER membrane localization of human *XBP1* U is independent of not only the 3′ UTR of *XBP1* U but also the hIRE1 foci^36^,^37^. However, the oligomerization into foci also holds true for the activation of mammalian IRE1^38^. The higher-order dynamic oligomerization of hIRE1 takes places upon ER stress, correlating with the onset of hIRE1 phosphorylation and RNase activity, and the dis-association of the foci after prolonged ER stress attenuates the UPR signaling by introducing hIRE1 de-phosphorylation and decline in RNase activity^38^. Thus, the mechanistic feature of higher-order IRE1 oligomerization emerges as a conserved mechanism of IRE1 signaling in yeast and metazoan cells.

Clearly, the homotypic interaction of the stress-sensing luminal domain of IRE1p is a key to the initiation of IRE1p signaling^31^,^33^,^34^. In accompany with this, the juxtaposed kinase domain on the cytoplasmic side of the ER is *trans*-autophosphorylated^31^,^38^,^39^,^40^,^41^. Based on the crystal structure of the dual catalytic region of IRE1p, the *trans*-autophosphorylation promotes nucleotide binding to a conventional nucleotide-binding cleft (the kinase active pocket)^27^. The resulting phosphorylation facilitates the back-to-back active dimerization of the cytosolic domains, which is further reinforced by higher-order oligomerization^32^,^33^,^34^, juxtaposing the RNase active sites and orienting the relevant residues for catalysis^33^,^38^. Based on this model, three key sites or molecular events have been employed to modulate the IRE1 activity: the nucleotide-binding pocket, the active dimerization and oligomerization (e.g. the dimer-interface pocket), and the RNase catalytic site^6^. Considering the central role of nucleotide binding in IRE1 activation, the hydrophobic kinase pocket has been shown to be engaged by ATP competitive inhibitors, such as APY24, Sunitinib, and CDK1/2 inhibitor III, to activate the RNase via stabilizing the active open conformation of the kinase that favours self-association of IRE1p^5^,^33^,^42^. Similarly, small molecular specific inhibitors, such as 4μ8C and STF-083010, have also been found to selectively modulate the activity of human IRE1’s RNase by forming an unusually stable Schiff base with lysine 907 4,6,43,44.

In this study, we studied divergence and conservation of the major branch of the UPR signaling pathway across kingdoms. Although plant bZIP60 has an ability to functionally substitute for HAC1p in yeast^26^, the products of the IRE1-*bZIP* duet have evolutionarily diverged. By contrast, the upstream component of this branch shows a high degree of conservation, including the mechanistic feature of IRE1 activation and the action mechanism of the dual cytosolic effectors. Our data shed new insights into the molecular aspects of divergence and conservation of the major UPR pathway branch across eukaryotes.

## Results

### Unlike bZIP60, XBP1 is not Functionally Complementary with HAC1p in Yeast

**Figure 1:**
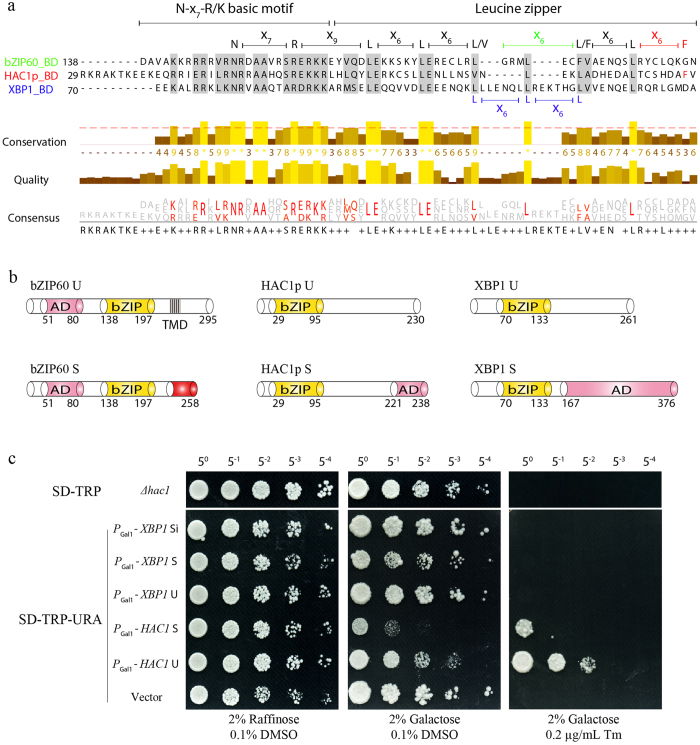
XBP1 is not Functionally Complementary with HAC1p in Yeast. (**a**) The conservation and divergence of the bZIP domain between HAC1p, bZIP60, and XBP1. The residues with a conservation threshold of >8 (red dashed line) were shaded in grey. The conservation quality and consensus sequence were given below with a histogram. A schematic of the bZIP domain consensus was shown above by extremely conserved residues and distance. Note that the N-x_7_-R/K basic motif is conserved in HAC1p, bZIP60 and XBP1. The zipper motif consists of five heptad Leucine-repeats in XBP1, whereas four in HAC1p and bZIP60 with and without interruption, respectively. (**b**) Although the AD locus in bZIP60 is different from that in HAC1p, their AD domains share a high identify in amino acid sequence^26^. However, the ADs of XBP1 S and HAC1p S are greatly different in length and in amino acid sequence, even though they both result from the unconventional splicing and have the same locus. (**c**) Functional testing of XBP1 in CRY1 Δ*hac1*::TRP strains. The untransformed or transformed yeast cells with empty CEN-ARS plasmid or plasmid harboring *HAC1* S, *HAC1* U, *XBP1* U, *XBP1* S and *XBP1* Si were normalized to an OD_600_ = 0.3 after 8 h of growth in raffinose-containing media, and 5-fold serial dilutions were spotted on raffinose and galactose-containing plates supplemented with 0.1% DMSO (control) or 0.2 μg/mL Tm. Plates were photographed after 2–3 days at 30 °C.

Recently we have shown that the spliced bZIP60 of plants can successfully substitute for HAC1p in yeast by rescuing the ER-stress sensitive phenotype of the *HAC1*-deficient yeast mutant^26^. To further examine whether this yeast gene could be functionally replaced by *XBP1*, the human mRNA part of the IRE1-mediated UPR arm^5^,^6^, we designed three types of *XBP1*, i.e., *XBP1* U, *XBP1* S and *XBP1* Si (Supplementary Fig. S1). cDNAs of HAC1p U, HAC1p S and *XBP1* variants were expressed in a *HAC1*-deficient yeast strain (CRY1 Δ*hac1p*::TRP)^45^,^46^ using a CEN-ARS plasmid containing a GAL1 inducible promoter. In accordance with the previous reports^11^,^26^,^47^, the constitutive expression of HAC1p U and HAC1p S compromises yeast growth under normal conditions (Fig. 1c). However, such a phenotype was not observed in yeast expressing XBP1 U, XBP1 S or XBP1 Si (Fig. 1c). Under 0.2 μg/mL tunicamycin (Tm) treatment, the Δ*hac1p*::TRP cells displayed a complete growth arrest, which could be rescued partially by the expression of HAC1p U and HAC1p S^26^, but not by that of XBP1 U, XBP1 S or XBP1 Si (Fig. 1c). These results suggest that HAC1p is not functionally replaceable with its human counterpart.

### No Inter-species Interaction among bZIP60, HAC1p and XBP1

**Figure 2:**
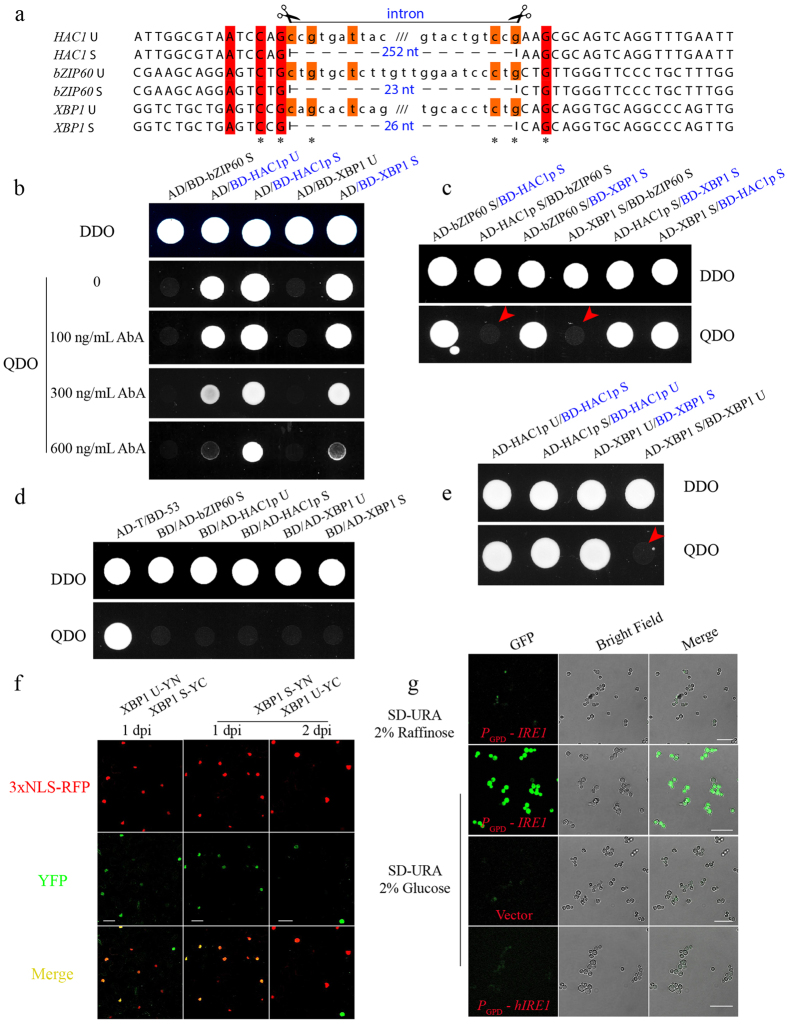
The Divergence among HAC1p, bZIP60 and XBP1. (**a**) Nucleotide sequence alignment of the twin hairpin loop in un-spliced and spliced *bZIP60, HAC1* and *XBP1* cDNA. Each of the two loops contains three conserved bases (asterisks). Scissors define the unconventional introns, and the numbers in blue indicate the intron length in each mRNA. (**b**) Self-activation of bZIP60 S, HAC1p U, HAC1p S, XBP1 U and XBP1 S fused to a Gal4 BD motif was tested in QDO media supplemented with different concentrations of AbA. Note that HAC1p U, HAC1p S and *XBP1* S (colored in blue) showed a strong self-activation. (**c**) bZIP60 S did not interact with HAC1p S and XBP1 S in yeast (red arrows, no yeast growth on QDO media). Fusion proteins colored in blue indicated the auto-activation, as shown in (**b**). (**d**) Self-activation of bZIP60 S, HAC1p U, HAC1p S, XBP1 U and XBP1 S fused to a Gal4 AD motif was tested on QDO media. The AD-T and BD-53 combination was utilized as a positive control, whereas the AD-T and BD-Lam set was used as a negative control (data not shown). (**e**) The un-spliced XBP1 did not interact with the spliced XBP1 in the Y2H assay (red arrow). Fusion proteins colored in blue indicated the auto-activation, as shown in (**b**,**c**). (**f**) XBP1 U and XBP1 S were transiently co-expressed in leaves of *N. benthamiana* along with a 3xNLS-RFP nucleus marker. The BiFC signal (in green) showing positive interactions between XBP1 U and XBP1 S was monitored at 1 or 2 day post agro-infiltration (dpi). Bars = 50 μM. (**g**) Functional complementation was carried out on CRY1 Δ*ire1*::KanMX6 strains with an integrated pRS304 4xUPRE-GFP reporter. The yeast cells transformed with CEN-ARS plasmids expressing IRE1p showed the activated UPR in the presence of 2% glucose under ER stress induced by 2 mM DTT, compared to the raffinose-grown yeast, whereas the cells expressing hIRE1 showed no activated UPR. Bars = 25 μM.

Sequence comparison showed that XBP1 S has a longer transcription activation domain (AD) than bZIP60 and HAC1p S (Fig. 1b). In addition, their DNA binding domains (BD) harbor differentiated characteristic Leucine-repeat motifs (Fig. 1a). As the leucine zipper is critical for transcription factor dimerization upon binding to target DNA^48^,^49^, we presumed that this motif may have a role in determining inter-species interactions, thereby functional interchangeability. To test this presumption, we conducted yeast two-hybrid (Y2H) assays using the Gold yeast strain. bZIP60 U was not included in the Y2H assay as it is an ER transmembrane protein^15^,^26^,^50^, whereas HAC1p U and XBP1 U were included due to their distribution in the nucleus *in planta* or in yeast (Supplementary Fig. S2). As shown in Fig. 2, the fusion of bZIP60 S or XBP1 U to a Gal4 BD motif could not activate the expression of reporter genes in the presence of the AD motif (Fig. 2b). However, the strong autonomous activation of the reporter genes was evident when the AD motif was co-expressed with the fusion protein of HAC1p U, HAC1p S or XBP1 S with the Gal4 BD motif (in blue), as indicated by yeast growth even under high concentrations of AbA (Fig. 2b). This self-activation prevented us from examining the inter-species interaction between HAC1p S and XBP1 S (Fig. 2c, in blue) and the interaction of HAC1p S and HAC1p U (Fig. 2e, in blue). The expression of all these five proteins fused to the Gal4 AD motif (as prey) could not lead to the activation of *HIS3* and *ADE2* reporter genes (Fig. 2d). Therefore, by ruling out the self-activation caused by BD-HAC1p U, BD-HAC1p S and BD-XBP1 S (in blue), we concluded that bZIP60 S did not exhibit the inter-species interaction with HAC1p S or XBP1 S (Fig. 2c, red arrows), and that the un-spliced and spliced *XBP1* did not interact in the Y2H assay (Fig. 2e, red arrow).

We further examined the interaction *in planta* via bimolecular fluorescence complementation (BiFC), wherein they were fused with both the N-terminal 174-aa portion (YN) and the C-terminal 65-aa part (YC) of the yellow fluorescence protein (YFP)^26^. Co-expression of YN- and YC-fused bZIP60 S, HAC1p S or XBP1 S resulted in strong YFP fluorescence in the nucleus, indicated by the 3xNLS-RFP nuclear marker, suggesting that the self-interactions of bZIP60 S, HAC1p S or XBP1 S occurs inside the nucleus *in planta* (Supplementary Fig. S2). By contrast, no yellow fluorescence signals were evident from plant cells co-expressing combinations of heterogeneous proteins, e.g., bZIP60 S and HAC1p S, HAC1p S and XBP1 S, or bZIP60 S and XBP1 S (Supplementary Fig. S2), showing no detectable inter-species interactions. Taken together, the data obtained in yeast and *in planta* suggest that the bZIP orthologues only allow for self-dimerization over the time course of evolution. It should be pointed out that we did find that XBP1 U interacted with *XBP1* S in the nucleus of plant cells (Fig. 2f), in contrast to the negative interaction observed in yeast (Fig. 2e). The reason that caused this discrepancy is to be understood.

### The mRNA Substrate for IRE1-mediated Unconventional Splicing is Species-specific

As mRNA substrates of unconventional splicing, their functional divergence may be rendered not only by non-permission of inter-species interactions, but also by the target specificity. This assumption is supported by the published data that *HAC1* U mRNA is not edited by *Arabidopsis* IRE1A and IRE1B in yeast or in protoplasts^26^,^51^, and *bZIP60* U mRNA cannot be spliced in yeast^26^. We thus further examined the underlying mechanisms of the substrate specificity of unconventional splicing. As shown in Fig. 2, the twin loops of *bZIP60* and *XBP1* have evolutionarily diverged in length from those in *HAC1*. Moreover, except the three conserved nucleotides (denoted by asterisks) contained in each loop, the two kissing loops are apparently non-conserved among the three mRNAs (Fig. 2a), implying that the unconventional splicing may be species-specific. We speculated that the specificity is reciprocal. To prove this, we further express human IRE1α (afterwards abbreviated hIRE1) in yeast to see if *HAC1* could be spliced. The CRY1 Δ*ire1*::KanMX6 yeast cells, with an integrated pRS304 4xUPRE-GFP reporter^45^,^46^, were transformed with CEN-ARS plasmids expressing yeast IRE1p and hIRE1 under the control of a GPD promoter. Under ER stress, the induction of IRE1p by glucose led to a remarkable UPR activation, compared to the yeast grown under raffinose containing media, indicating that *HAC1* was spliced (Fig. 2g). However, hIRE1 could not activate the UPR in yeast (Fig. 2g), demonstrating that hIRE1 cannot process *HAC1* in yeast.

### *Arabidopsis* IRE1B, rather than IRE1A and hIRE1, Clusters in Yeast

**Figure 3:**
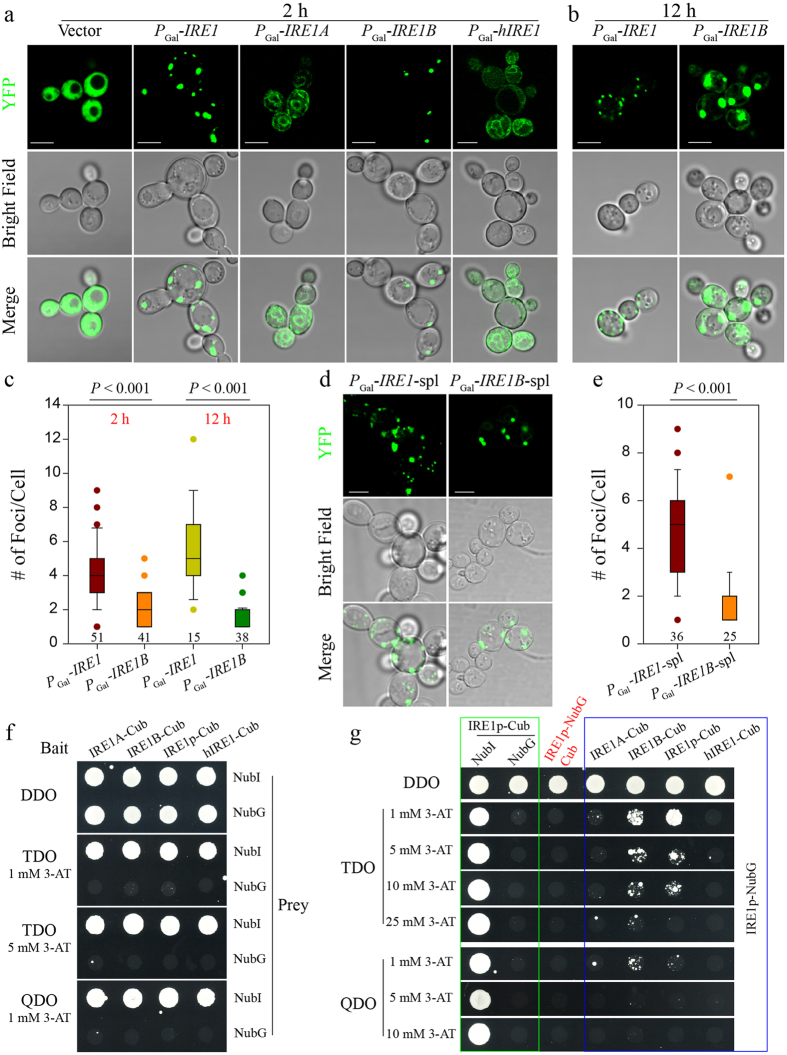
IRE1B Forms Foci in Yeast and Interacts with IRE1p. (**a**) The formation of foci by IRE1p and IRE1B, rather than free YFP, IRE1A and hIRE1, under ER stress induced with 10 mM DTT for 2 h. Bars = 5 μM. (**b**) The IRE1p- and IRE1B-formed foci under ER stress induced with 10 mM DTT for 12 h. Bars = 5 μM. (**c**) Box plots depict the number of IRE1p- and IRE1B-formed foci per cell after 10 mM DTT treatment for 2 and 12 h. Statistical significance of the difference between IRE1p- and IRE1B-induced foci was analyzed by unpaired two-tailed Student’s test. The number of cells calculated was indicated below each box. (**d**) The formation of foci by the spl domains of IRE1 and IRE1B under ER stress induced with 10 mM DTT for 2 h. Note that the spl domains can be referenced in Supplementary Fig. S3. Bars = 5 μM. (**e**) Box plots depict the number of foci formed by the spl domains of IRE1p and IRE1B per cell after 10 mM DTT treatment for 2 h, with the same statistical analysis as in (**c**). The number of cells tested was indicated below each box. (**f**) The yeast strain NMY51 was co-transformed with pBT3-SUC bait plasmids expressing the indicated IRE1 orthologues and pOST-NubI or pPR3-C prey constructs. The encoded IRE1A, IRE1B, IRE1p or hIRE1 were fused to the C-terminal half of ubiquitin (Cub). The pOST-NubI and pPR3-C constructs encoding wild type Nub (NubI) and a point mutant of the N-terminal half of ubiquitin (NubG) were used as positive and negative controls, respectively. Yeast colonies were plated on permissive DDO media and selective media TDO and QDO in the presence of 3-AT. (**g**) Parts of the data in a green box were from (**f**) and repeated for showing no self-activation caused by the expression of IRE1p as a bait in selective media. The expression of IRE1p as a prey in the presence of Cub did not cause self-activation (marked in red). Therefore, it was concluded that IRE1p interacted not only with itself but also with IRE1B, and no interaction could be found between IRE1A and IRE1p or hIRE1 and IRE1p, shown in a blue box.

Previous studies demonstrated that IRE1 clustering to foci is a prerequisite for substrate mRNA recruitment and splicing in yeast and human cells^32^,^34^,^38^. To know how much the higher-order oligomerization of IRE1 is conserved, we examined and compared the foci of *Arabidopsis* IRE1A and IRE1B, yeast IRE1 and human hIRE1 tagged by YFP at their N- or C-termini (data not shown) in yeast. The free YFP (empty vector), served as a control, showed an even distribution throughout the cell under ER stress (Fig. 3a). As expected, we observed the typical IRE1p loci after DTT treatment for 2 h, consistent with the previous observations about the distribution pattern, size and number of foci (Fig. 3c)^32^,^34^. Strikingly, IRE1B developed discrete foci in yeast, with a similar size and distribution pattern to IRE1p, despite a significant reduction in the number of foci (Fig. 3a,c, *P* < 0.001). IRE1A and hIRE1, however, distributed diffusely throughout the ER, apparently lacking the ability to form the IRE1p-like foci (Fig. 3a). The foci formed by IRE1p and IRE1B presented contrasting dynamics during the time course of ER stress: the IRE1p foci became smaller whereas the IRE1B foci were enlarged after 12 h treatment with 10 mM DTT (Fig. 3b). Moreover, IRE1B accumulated in the ER upon prolonged ER stress, but IRE1p did not (Fig. 3b). The number of IRE1p or IRE1B foci per cell remained similar during the time course of the experiment (Fig. 3c).

Since the kinase and RNase domains are dispensable for IRE1p clustering and mRNA recruitment in yeast^32^, we examined the features of the IRE1B-formed heterogeneous foci by deletion of its kinase and RNase domains. The N-terminus of IRE1 without kinase and RNase domains contains the signal peptide, sensor, transmembrane and linker domains^32^,^34^, and, for convenience of description, was abbreviated to spl (Supplementary Fig. S3). It was found that the foci could be formed by IRE1B-spl, similar to those by IRE1p-spl (Fig. 3d). Moreover, the size, distribution and number of foci of IRE1B-spl were similar to those of full-length IRE1B (Fig. 3a,d). However, the number of foci of IRE1B-spl was significantly fewer than that of IRE1p-spl, which was the same as described for full-length IRE1p versus IRE1B (Fig. 3c,e).

It has been reported that oligomeric assembly of the ER-luminal domain is sufficient to drive IRE1 clustering in yeast^32^, and dimerization to produce a composite groove extending across the luminal domains of the two IRE1p is required for oligomerization^40^,^46^. Therefore, we examined the homo- and hetero-interaction of IRE1A and IRE1B to understand the underlying mechanism by which two *Arabidopsis* IRE1 homologues displayed contrasting abilities to form foci in yeast. We have recently shown the homo-interaction of full-length IRE1A and IRE1B^26^. We thus focused on the interaction of the luminal domains of IRE1A and IRE1B. BiFC assays showed that both luminal domains of IRE1A and RIE1B, i.e., IRE1A-SPS and IRE1B-SPS (the signal peptide and sensor domain), displayed not only homo-interactions but also hetero-interactions (Supplementary Fig. S3). Moreover, the BiFC signal showed a perfect co-localization with the KDEL-mCherry, an ER-labelled marker, rather than with the 3xNLS-CFP localized in the nucleus (Supplementary Fig. S3). Then, we examined the interaction of the core luminal domains of IRE1A and IRE1B, i.e., IRE1A-S and IRE1B-S (the sensor domain), by removing their SP domains (Supplementary Fig. S3). The core luminal domain of IRE1A without the SP domain (IRE1A-S) showed the ability to self-interact, as the majority of cells (90%, n = 10) displayed the BiFC signal in both the cytoplasm and nucleus (Supplementary Fig. S3). YFP fluorescence resulting from the homo-interaction of IRE1B-S was also evident: about 50% cells (n = 22) displaying the BiFC signal in both the cytoplasm and nucleus (Supplementary Fig. S3). In reciprocal BiFC assays, nearly all cells tested showed BiFC signals in the cytoplasm, indicating IRE1A-S and IRE1B-S heterogeneously interacted (Supplementary Fig. S3). Like IRE1A-S and IRE1B-S, full-length IRE1A and IRE1B also hetero-interacted in reciprocal BiFC assays (Supplementary Fig. S3).

Therefore, the self-interaction is not the determinant to the heterogeneous foci of IRE1A and IRE1B in yeast. We, thus, attempted the inter-species interaction of the four IRE1s. In a split-ubiquitin Y2H system, the bait self-activation was firstly tested with the bait constructs pBT3-*IRE1A*, pBT3-*IRE1B*, pBT3-*IRE1* and pBT3-*hIRE1*, paired with the pOST-NubI (positive) and pPR3-C (negative) prey constructs. As shown (Fig. 3f,g, green box), the four bait constructs combined with pOST-NubI led to yeast growth in all selective media tested, whereas no yeast colonies could grow when the negative prey construct was co-transfected, showing no auto-activation of all four bait constructs. We further found that the prey construct pPR3-*IRE1* could not cause the auto-activation in the presence of the empty bait construct pBT3-C (Fig. 3g, red). Therefore, we could use the combination of pBT3-*IRE1A*, pBT3-*IRE1B*, pBT3-*IRE1* or pBT3-*hIRE1* with the pPR3-*IRE1* for examining the possible interactions. As expected, IRE1p displayed a self-interaction in TDO media even with 10 mM 3-AT and in QDO media with 1 mM 3-AT (Fig. 3g, blue box). Interestingly, plant IRE1B was found to interact with IRE1p in the same selective media (Fig. 3g, blue box). No salient interaction, however, could be detected between IRE1A and IRE1p or between hIRE1 and IRE1p (Fig. 3g, blue box).

### Conservation of Kinase and RNase Domains of IRE1 Orthologues

**Figure 4:**
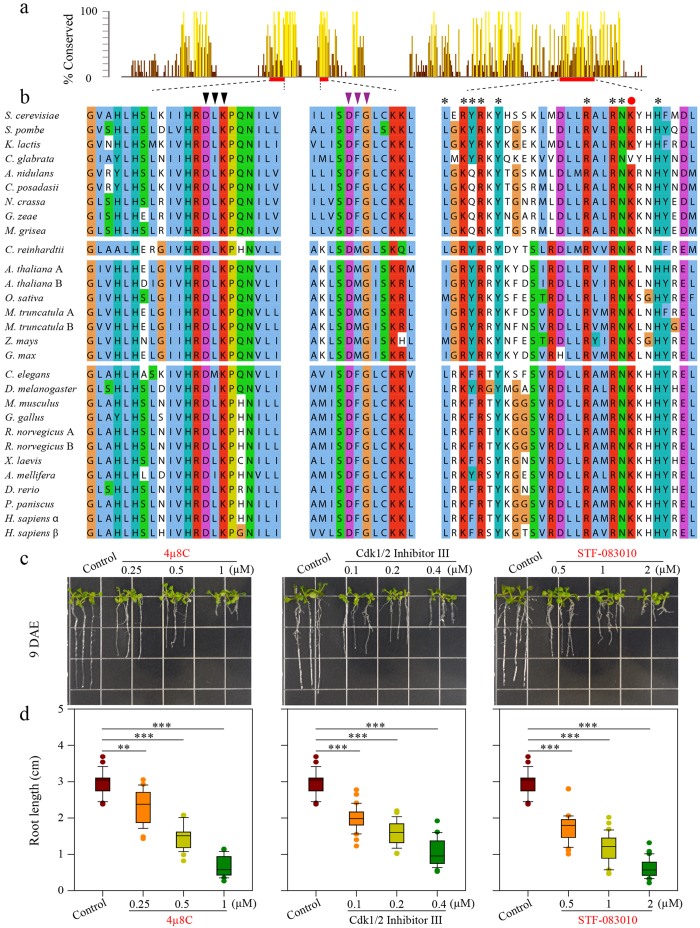
The Kinase and RNase Domains of IRE1 Orthologues are Conserved among Fungi, Plantae and Animalia. (**a**) Conservation of the cytosolic domains of IRE1 orthologues. A bar diagram (a panel of Supplementary Fig. S5) displays the relative conservation of cytosolic domains of IRE1 homologues from fungi as well as orthologues from *C. reinhardtti* and plant and animal species. The key regions of nucleotide binding pocket, DFG kinase motif, and RNase inhibitor binding site were marked with a red box, whose sequence alignments were shown in **(b)**. (**b**) Black triangles denote the key residues in the hydrophobic nucleotide binding pocket, purple triangles indicate the DFG kinase motif^5^,^52^, and starts and red solid circle highlight the key residues in RNase domain for forming a hydrophobic pocket for docking of IRE1 inhibitor III, which covalently targets hIRE1 K907 (red solid circle) via Schiff base formation^6^. (**c**) The root phenotype of *Arabidopsis* seedlings treated with IRE1 inhibitors. Nine-day-old Col-0 seedlings grown on vertical one-half-strength MS media supplemented with 0.1% DMSO (solvent control) or various concentrations of 4μ8C (left), CDK1/2 Inhibitor III (middle), and STF-083010 (right). DAE, days after emergence. (**d**) Primary root length of 9-d-old seedlings was measured. Box plots summarize results from 23–31 independent replicates and show the median (horizontal line within the box), extent of the 1^st^ to 3^rd^ quartile range (box), values extending to 1.5 times the interquartile range (whiskers), and outliers (circles). **P < 0.01, ***P < 0.001, unpaired two-tailed Student’s test.

A phylogenetic analysis of 29 IRE1 orthologues that were retrieved from GeneBank using IRE1p as a query sequence clustered them into three major groups, matching to the three kingdoms *Animalia, Fungi* and *Plantae* (Supplementary Fig. S4), suggesting IRE1 is conserved within each kingdom. Moreover, the IRE1 cytosolic domains are more conserved across species than the core luminal domains (Supplementary Fig. S5). As shown in Fig. 4, besides the conserved DFG kinase motif (Fig. 4b, middle panel, purple triangles), the kinase domain of IRE1 contains a strictly conserved hydrophobic region that is crucial for binding nucleotides, and key residues (D, L, and K, indicated by black triangles) for forming the hydrophobic pocket are almost identical in all tested species^5^,^52^. Moreover, the other hydrophobic pocket formed in the RNase domain that is important for RNase activity is also highly conserved, and the RNase enzymatic active sites are nearly identical (denoted by asterisks)^6^.

It has been proven that the IRE1 Inhibitor III, including 4μ8C and STF-083010, inactivates RNase activity by direct binding to the conserved lysine (K, red circle, Fig. 4b, right panel), whereas the type I kinase inhibitors, including sunitinib and CDK1/2 inhibitor III, engage the conventional nucleotides-binding site in a manner compatible with the formation of back-to-back dimers, thereby stimulating IRE1 nuclease activity^5^,^33^,^43^. It is tempting to speculate that the action mechanism of the cytosolic domain may be evolutionarily conserved across kingdoms. To test this assumption, we examined whether the IRE1 inhibitors that work on human hIRE1 or yeast IRE1 can inhibit plant IRE1s. It has been reported that an *Arabidopsis* double mutant of *IRE1A* and *IRE1B* exhibits a short-root phenotype (Supplementary Fig. S6)^53^,^54^,^55^. As shown in Fig. 4, compared with the control (0.1% DMSO), the root elongation of wild-type *Arabidopsis* was significantly inhibited in the presence of 4μ8C in a dose-dependent manner (**P < 0.01, ***P < 0.001). The effect of STF-083010 on root growth was consistent with that of 4μ8C (Fig. 4c,d), supporting the notion that 4μ8C and STF-083010 act on the highly conserved lysine residue to destroy RNase activity^6^. Administrating CDK1/2 inhibitor III to *Arabidopsis* also induced a short-root phenotype, analogous to that resulting from treatment of 4μ8C or STF-083010 (Fig. 4c,d). These results suggest that both kinase and RNase domains are indispensable for root growth. To prove the specific effects of the inhibitors on IRE1s *in planta*, we also applied 4μ8C to the double IRE1A and IRE1B mutant *ire1a-2 ire1b-4*. It was found that the root growth of *ire1a-2 ire1b-4* could not be further inhibited by 0.5 μM 4μ8C (Supplementary Fig. S6), indicating the action specificity of inhibitors on IRE1s *in planta*. It should be pointed out that the inhibition of root growth by IRE1 inhibitor could be partially ascribed to the reduced cell number in the root meristem (Supplementary Fig. S6).

## Discussion

The IRE1-*bZIP* pathway is recognized as the most ancient and conserved branch of the UPR^3^,^7^,^10^. However, virtually little is known about the degree of its conservation across kingdoms. Following the establishment of plant bZIP60 as a swappable counterpart of HAC1p in yeast^26^, we further evaluated the degree of UPR diversification and the conserved underpinnings of the IRE1-*bZIP* branch across eukaryotes.

HAC1p in yeast, bZIP60 in *Arabidopsis* and *XBP1* in human, belonging to the bZIP family of transcription factors, are characterized with a strictly conserved N-x7-R/K DNA binding motif and a well-organized leucine zipper domain (Fig. 1a)^49^. As unconventional splicing mRNA substrates, they are positioned at the same signaling node in the UPR branch, to couple perturbed protein-folding homeostasis to redirecting transcriptional programs to mitigate ER stress^3^,^14^,^17^. In a recent work, we have demonstrated that the nucleus-located bZIP60 is functionally swappable with HAC1p in yeast^26^. However, in contrast to the plant counterpart, the heterogeneous expression of three XBP1 variants failed to compensate for the loss-of-function of HAC1p in yeast (Fig. 1c). It is therefore reasonable to conclude that there exists not only functional conservation (HAC1p and bZIP60) but also evolutionary divergence (HAC1p and XBP1) in this oldest UPR branch.

To gain a better understanding of evolutionary divergence, we looked at the AD domain of the three transcriptional factors. In contrast to bZIP60 U that includes an AD domain at its N-terminus, HAC1p and XBP1 gain the AD domain at their C-termini only after the unconventional editing (Fig. 1b)^13^,^21^,^56^. Although the localization of AD motifs in bZIP60 and HAC1p S is quite different, they have a high degree of similarity in length and sequence^26^. However, the AD domain in XBP1 S is 10-fold longer than that in HAC1p S or bZIP60 (Fig. 1b), and the AD sequence of *XBP1* S is highly diverse from that of HAC1p or bZIP60. Moreover, although the N-x7-R/K basic motif in the BD domain is highly conserved among HAC1p, bZIP60 and XBP1, the heptad repeat of leucine (leucine is replaceable by isoleucine, valine, phenylalanine or methionine) of the leucine zipper domain is evolutionarily diverse, e.g., five clustered heptad leucine-repeats in XBP1 and four in bZIP60 without interruption, in contrast to four in HAC1p with a L/V-x4-L motif insertion (Fig. 1a). Based on these differences, we proposed that the way to regulate UPR-responsive genes by XBP1 in human has evolved divergently from that by HAC1p in yeast or that by bZIP60 in *Arabidopsis*.

The ability of bZIP transcriptional factors to form homo- or hetero-dimers is determined by the electrostatic attraction and repulsion of polar residues flanking the surface of amphipathic helix determined by clustered Leucine-repeats^48^,^49^. Therefore, the differential Leucine-repeats in HAC1p, bZIP60 and XBP1 may have been evolutionarily established to tune interaction specificity in their own transcriptional programs. Our recent work has shown that bZIP60 S displays a strong self-interaction in the Y2H assay^26^. The self-interaction also holds true for HAC1p S and XBP1 S, revealed by the BiFC assays *in planta* (Supplementary Fig. S2). However, bZIP60 S did not interact with HAC1p S and XBP1 S in yeast (Fig. 2b–d, red arrows), which was further confirmed by the BiFC assays *in planta* (Supplementary Fig. S2). Though we could not determine if HAC1p S interacts with XBP1 S in yeast due to the auto-activation of their bait fusion proteins (Fig. 2a,c), our BiFC assay *in planta* uncovered no inter-species interaction between HAC1p S and *XBP1* S (Supplementary Fig. S2). Based on these data, we conclude that the inter-species hetero-interaction is not permitted, indicating an important aspect of evolutionary divergence among HAC1p, bZIP60 and XBP1. In human cells, XBP1 U can interact with XBP1 S, and the complex is exported to the cytoplasm for proteasome-dependent degradation because a nuclear export signal and a degradation domain are present in the C-terminus of XBP1 U^12^,^23^. As a result, XBP1 S action is shut down during the later phase of ER stress, and XBP1 U is thus regarded as an inhibitor of the UPR in higher eukaryotes^12^,^23^,^57^. In this study, XBP1 U was found to interact with *XBP1* S *in planta*, but not in yeast (Fig. 2b,d,e, red arrow; Fig. 2f). Although XBP1 U or XBP1 S *in planta* and in yeast displayed the same distribution pattern as in human cells (Supplementary Fig. S2)^23^,^26^, we could only observe the complex of XBP1 U and XBP1 S in the nucleus *in planta* (Fig. 2f). This is possibly due to the proteasome-mediated rapid degradation of the heterogeneous XBP1 U-XBP1 S complex in the cytoplasm, as previously described in human cells^23^.

The cytosolic portion of IRE1p possesses the ability to edit a synthetic RNA substrate containing the *XBP1* splicing site *in vitro*, and a hybrid IRE1 containing the luminal, transmembrane and juxta-membrane domains of hIRE1 and the kinase and endonuclease domains of IRE1p can execute the unconventional splicing of *XBP1* mRNA in mammalian cells^5^,^33^. However, we found that IRE1p failed to edit *XBP1* mRNA in yeast (Supplementary Fig. S2), implying that *XBP1* mRNA is recruited in a way different from *HAC1* mRNA. In addition, in a reciprocal assay in which hIRE1 was expressed in a UPR-reporting yeast stain, hIRE1 was unable to splice *HAC1* mRNA and activate UPR in yeast (Fig. 2g). This is in line with the finding that plant IRE1A or IRE1B cannot carry out the unconventional splicing of *HAC1* mRNA in yeast^26^ and in *Arabidopsis* protoplasts^51^. This marked contrast in the unconventional editing of HAC1 and XBP1 could not be completely attributed to the different IRE1 activation mechanisms (see discussion below). A comparison of the sequences around the splicing sites of *HAC1, bZIP60* and *XBP1* revealed that, except the strictly conserved nucleotides that are necessary for unconventional splicing^21^,^22^,^23^, these mRNAs do not share a high sequence identity (Fig. 2a, conserved nucleotide marked by asterisk). Moreover, the intron in *HAC1* is around 10-fold longer than that in *bZIP60* or *XBP1*, representing a distinctive divergence in substrate itself, and the base-paring interaction between the intron and leader sequence is only found in *HAC1*^22^,^35^. Thus, *bZIP60* and *XBP1* are remarkably divergent from *HAC1*, and it is not surprising to observe that plant IRE1s and human hIRE1 failed to edit *HAC1* in yeast.

Evolutionary divergence of the unconventional splicing substrates suggests, on one hand, a set of transcriptional targets to be specifically regulated in their own species, and implies the diverse mechanisms by which IRE1 is activated, on the other hand. Indeed, IRE1p clustering in the ER membrane is indispensable for *HAC1* mRNA recruitment and splicing in yeast (Fig. 3a,d)^31^,^32^,^34^, and this feature of IRE1 activation in response to ER stress is conserved in metazoan cells^38^. In our attempt to compare the clustering of IRE1 orthologues from three species in yeast cells, we surprisingly found that hIRE1 was unable to form foci in yeast (Fig. 3a). IRE1p can be directly bound by an unfolded protein as ligand, and the unfolded protein binding to IRE1p is the single and sufficient step for activation of the UPR in yeast cells^45^,^46^. Nevertheless, the luminal regions of hIRE1 do not interact with unfolded proteins in a cell-free system^58^, and, unlike yeast IRE1p, the MHC-like groove in the crystal structure of human IRE1 is too narrow for peptide binding^59^. Therefore, hIRE1 signaling cannot be initiated in yeast, and consistently hIRE1-mediated heterogeneous foci cannot be induced either.

We also surprisingly found that two IRE1 homologues from *Arabidopsis* displayed contrasting abilities to oligomerize in yeast cells: IRE1B could form typical foci but IRE1A could not (Fig. 3a,c). Strikingly, removal of the entire kinase and RNase domains of IRE1B did not affect the formation of foci (Fig. 3a,d), which is consistent with the findings obtained from the similar work done with IRE1p (Fig. 3a,d)^32^. Prolonging ER stress caused not only the enlargement of IRE1B foci but also changed its distribution pattern (Fig. 3b). In the case of IRE1p, although the number of foci per cell during the time course of ER stress remained similar, the size of IRE1p foci was reduced at 12 h of ER stress (Fig. 3a–c). The similar size change was also found for hIRE1 foci in human cells^38^. The dissolution of hIRE1 clusters is accompanied with hIRE1 de-phosphorylation, reduction of endoribonuclease activity, and arrest of *XBP1* mRNA splicing, indicating that hIRE1 signaling enters a refractive state and directs the UPR from the cytoprotective phase to the apoptotic mode^38^. At this point, a similar timer mechanism may be employed to govern IRE1p signaling in yeast cells, if ER stress remains unmitigated. The enlargement of IRE1B clusters after prolonged ER stress in yeast presumably indicates the status of IRE1B beyond the control by the timer.

In our recent report, we have shown that both IRE1A and IRE1B can be homo-dimerized^26^. In this study, we further demonstrated that IRE1A can also interact with IRE1B (Supplementary Fig. S3). Moreover, the luminal domains of IRE1A and IRE1B with or without the signal peptide displayed not only homo-interactions but also hetero-interactions (Supplementary Fig. S3). The ability to homo-dimerize may suggest that both IRE1A and IRE1B are able to cluster into foci *in planta*, like IRE1p in yeast and hIRE1 in human cells^32^,^38^, whereas the biological significance of hetero-dimerization remains to be identified. It has been reported that IRE1A and IRE1B are functionally redundant at least in regulating organ-specific growth and editing *bZIP60* mRNA in response to abiotic and biotic stresses^15^,^26^,^28^,^54^,^55^. Apparently, all these findings support that IRE1A and IRE1B remain highly homologous in the time course of evolution. However, a closer look at IRE1 orthologues from an evolution point of view revealed that IRE1B has diverged from IRE1A at a relatively early stage (Supplementary Fig. S4). Interestingly, the divergence of IRE1B from IRE1A and other plant IRE1 homologues resembles that of IRE1p from other fungi IRE1 homologues (Supplementary Fig. S4). In this study, we also found that only IRE1B, rather than IRE1A and hRIE1, could interact with IRE1p (Fig. 3g). Based on these analyses, we propose that IRE1B is evolutionarily closer to IRE1p than IRE1A and hIRE1, and thus has an ability to form foci in yeast cells. It should be interesting to test whether IRE1B can adopt the IRE1p activation mechanism via direct binding to unfolded proteins, and whether IRE1B heterogeneous foci can entrap the *HAC1* mRNA substrate to the ER membrane in yeast cells.

From an evolution angle, the cytosolic domains of IRE1 homologues are much more conserved across eukaryotes than the luminal sensor domains (Supplementary Fig. S5). Particularly, the nucleotide binding pocket, DFG kinase motif and the putative RNase active motif^27^ are highly conserved in amino acid sequence among diverse species (Fig. 4a,b; Supplementary Fig. S5). Treatment with 4μ8C or STF-083010, two IRE1 inhibitors known to specifically inactivate the RNase activity of human IRE1^5^,^33^,^43^, induced a short-root phenotype (Fig. 4c,d), which is accordance with the root growth phenotype of the *Arabidopsis IRE1A* and *IRE1B* double mutant^53^,^54^,^55^. Moreover, the kinase specific inhibitor (CDK1/2 Inhibitor III) for IRE1p also displayed an inhibitory effect on root growth (Fig. 4c,d). Therefore, our data point to the requirement of both kinase and RNase of plant IRE1s for root development. This result is consistent with the finding that a single mutation in kinase or RNase fails to complement the stress-tolerance defect and short-root phenotype in the double mutant of *IRE1A* and *IRE1B*^54^. However, it should be pointed out that the short-root development observed in the presence of CDK1/2 inhibitor III might result from the stimulated RNase activity, considering the fact that CDK1/2 Inhibitor III is not only an inhibitor for the kinase activity of IRE1p but also a potent activator of IRE1p RNase^5^. Further studies using specific activator(s) of RNase are needed to clarify the unknown effect(s) of increased RNase activity *in planta*. Taken together, our findings suggest that the cytosolic domains of IRE1 orthologues are highly conserved, and there exist conserved mechanisms whereby chemical inhibitors bind to the dual functional domains, providing a simple platform for screening drugs targeted to the indicated sites in order to treat diseases.

**Figure 5:**
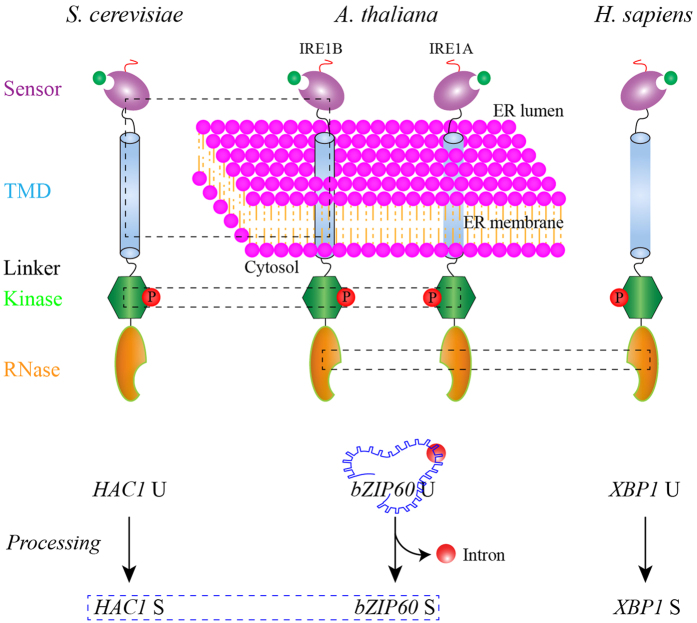
A Schematic Depicts the Conservation and Divergence of IRE1-*bZIP* Pathways. IRE1s from yeast, *Arabidopsis* and human are divided in sensor, TMD, linker, kinase and RNase domains (upper panel). Upon sensing the unfolded settings (indicated by green circle) by sensor domains, IRE1s were activated to process their mRNA targets (lower panel). Blue box (lower panel) represents the functional conservation between bZIP60 and HAC1p, as reported^26^. The red box in the lower panel indicates the divergence among *HAC1, bZIP60* and *XBP1*, demonstrated by non-permission of heterogeneous processing/interactions and by the inability of XBP1 to replace HAC1p in yeast. Black boxes (upper panel) indicate conservation found in IRE1 orthologues, including the heterogeneous formation of IRE-mediated foci, the interaction of IRE1B with IRE1p and the same molecular mechanism underlying responses of cytosolic domains of IRE1s to specific inhibitors. Red circles labelled with P indicate the phosphorylation of IRE1 orthologues in their kinase domains (upper panel), and a red circle indicates the unconventional intron in *bZIP60* U, which is omitted in *HAC1* U and and *XBP1* U (lower panel).

In this contribution, we provide evidence that, except the replicability of bZIP60 and HAC1p in yeast^26^, the products of the IRE1-*bZIP* duet among three eukaryote species evolutionarily differentiate (Fig. 5, red box). This may result from a transcriptional selection in the course of species evolution to meet the demanding of regulation fidelity of a set of specialized genes. By contrast, the upstream component of this branch showed a high degree of conservation among species tested, including the activation mechanisms of the ER transducer, the heterotypic interaction, and the action model of the cytosolic domains revealed by inhibitors assays (Fig. 5, black boxes). Taken together, our data reveal the molecular underpinnings of divergence and conservation of this oldest UPR pathway across eukaryotes, and shed new lights into its activation and action mechanisms.

## Methods

### Entry Vector Construction

Unless indicated otherwise, all DNA sequences were amplified by Phusion High-Fidelity DNA Polymerase (NEB, USA) using the primer sets listed in Supplementary Table S1 and Gateway technology (Invitrogen, USA) was employed to generate plasmids. The entry vectors bearing *HAC1* U, *HAC1* S, *bZIP60* U, *bZIP60* S, *IRE1A, IRE1B, IRE1* and *IRE1p* have been described^26^. The sequences of *XBP1* U, *XBP1* S, *XBP1* Si and *hIRE1* were synthesized by GeneScript (Piscataway, NJ) and were introduced into pDONR221 (Supplementary Fig. S1). The encoding regions of SPS domain, sensor domain, spl domain for each IRE1 orthologue were amplified from their entry vectors using the primers listed in Supplementary Table S1, then recombined into pDONR221 via the BP reaction (Invitrogen, USA). To highlight the nucleus, constructs bearing *35S::3xNLS-CFP* and *35S::3xNLS-RFP* were created based on the BP and LR reactions using the primers listed in Supplementary Table S1.

### Complementation Test in Yeast

The functional complementation assay was performed essentially as described in our recent report with minor modifications^26^. The donor vectors bearing *XBP1* U, *XBP1* S and *XBP1* Si were recombined into a CEN-ARS Gateway destination vector pAG416GAL-ccdB-HA, and the resulting constructs and the empty vector were transferred into the CRY1 Δ*hac1*::TRP strains, as described previously^26^. The transformed CRY1 Δ*hac1*::TRP yeast cells were grown to the mid-log phase at 28 °C in a 2x synthetic media lacking TRP and URA in the presence of 2% glucose, then switched into 2% raffinose containing media for 8 h to relief the glucose repression of Gal1 promoter. The raffinose-grown cells were collected and normalized (OD_600_ = 0.3), and 5-fold serial dilutions were spotted on the 2x SD-TRP-URA agar media containing 2% galactose or raffinose with or without 0.2 μg/mL tunicamycin (Tm) and incubated for 2–3 d at 28 °C. The un-transformed Δ*hac1* cells and the cells transformed with empty vector were used as negative controls, and HAC1p U and HAC1p S expressing vectors were used as positive references, as described^26^.

The heterogeneous UPR induction was tested on CRY1 Δ*ire1*::KanMX6 strains with an integrated pRS304 4xUPRE-GFP reporter, as described^26^,^45^. The cells were transformed with a CEN-ARS vector (pAG416GPD-ccdB-HA) bearing *IRE1* and *hIRE1* under the control of a GPD promoter. The transformed yeast cells were selected in the 2xSD media deficient in URA with 2% raffinose and confirmed by PCR. The positive colonies were cultured in 2xSD-URA media in the presence of 2% glucose or 2% raffinose. At the exponential growth stage, the yeast cultures were incubated with 2 mM DTT for 2 h and processed for microscopy to visualize the UPR reporter, as described in our previous report^26^.

### Yeast Two-Hybrid (Y2H) Assays

The Y2H assays, using the Y2H Gold yeast strain (Clontech), were performed as described previously^26^. Briefly, the donor vectors with cDNAs of *bZIP60, HAC1* and *XBP1* were recombined into pGBKT7-GW (bait) and pGADT7-GW (prey) vectors based on the LR reaction (Invitrogen, USA). The resulting constructs were transformed into the strain indicated above, and yeast cells were selected on a TRP- and LEU-deficient double dropout (DDO) media. Transformed colonies, confirmed by PCR and western blotting, were plated onto a HIS-, TRP-, LEU- and ADE-deficient quadruple dropout (QDO) media with the indicated concentrations of aureobasidin A (AbA) to test possible interactions.

The split-ubiquitin membrane Y2H assays were carried out on the NMY51 yeast strain as described previously^60^. The encoding regions of IRE1 homologues, which were amplified from their donor vectors using the primers containing *Sfi* I site listed in Supplementary Table S1, were sub-cloned into the bait vector pBT3-SUC and the prey vector pPR3-SUC. The yeast cells co-transformed with the indicated bait-prey constructs were transformed into the yeast cells, and selected as described above. Baits expressing IRE1 orthologues against empty prey vector (pPR3-C) or the reciprocal combinations were also transformed into yeast cells test potential bait and prey auto-activation. The combination of pOST-NubI with each IRE1 bait vector was used as a positive reference. Interactions were determined by yeast growth on a synthetic triple dropout (TDO) media deficient in HIS, TRP and LEU supplemented with 1 mM, 5 mM, 10 mM and 25 mM 3-Amino-1,2,4-Triazole (3-AT) and the QDO media in the presence of 1 mM, 5 mM, and 10 mM 3-AT. The experiments were repeated at least three times.

### BiFC Assays

The relevant entry vectors were cloned into the Gateway version of BiFC vectors to fuse the split YN and YC. The indicated combinations of resulting expression constructs were subjected to the agrobacterium-mediated transient expression system in *N. benthamiana*. To highlight the interaction place within cell, a nucleus indicator (35S::3xNLS-CFP or 35S::3xNLS-RFP) or/and a ER marker KDEL-mCherry were co-infiltrated with BiFC constructs. One or two days after transformation, BiFC fluorescence and their subcellular localizations were observed under a confocal microscope using a sequential scanning model, as described previously^26^. The experiments were repeated three times with similar results.

### Microscopy and Foci Imaging

Using the relevant entry constructs, the LR recombination was conducted onto Gateway vectors pAG423GAL-ccdb-EYFP and pAG423GAL-EYFP-ccdb, resulting in IRE1A, IRE1B, IRE1 and hIRE1 fused to YFP at their N-termini or C-termini, respectively. In the same way, their SPL domains were fused to YFP at either end. The resulting constructs and the empty vector were transferred into the CRY1 Δ*hac1*::TRP strains, and colonies were selected in a 2xSD-TRP-HIS in the presence of 2% glucose, as described above. After confirmation by PCR, the cells were cultured in the selective media with glucose, and switched into 2% raffinose containing media for 8 h, as described above. The raffinose-grown cells were switched into 2x SD-TRP-HIS media containing 2% galactose to induce the expression of the fusion constructs at 28 °C. After 8 h induction, the yeast cells were then incubated with 10 mM DTT for 2 h or 12 h and processed for microscopy to visualize IRE1 foci. YFP signal was captured under an inverted confocal microscope (TCS SP2, Leica, Germany) using a 514 nm excitation light and a 525–550 nm band-pass filter. The experiments were repeated three times with similar results.

### Root Growth and Drug Treatment

*Arabidopsis thaliana* used in this study is in the Columbia-0 (Col-0) background. Surface-sterilized seeds were kept at 4 °C for 3 d and directly plated onto dishes, as described^26^. For drug treatment, the plant growth media were supplied with 0.1% DMSO or 4μ8C (EMD Millipore), CDK1/2 Inhibitor III (EMD Millipore) and STF-083010 (Sigma) at the indicated concentrations. For drug dose screening, a preliminary estimation of the effects on root growth was carried out from a wider concentration range: 4μ8C (0.5 μM, 10 μM, 50 μM), CDK1/2 Inhibitor III (0.1 μM, 1 μM, 10 μM), and STF-083010 (1 μM, 10 μM, 50 μM). The normal plant growth conditions were set at 21 °C under a 16-h light/8-h dark cycle.

## Additional Information

**How to cite this article**: Zhang, L. *et al*. Divergence and Conservation of the Major UPR Branch IRE1-*bZIP* Signaling Pathway across Eukaryotes. *Sci. Rep.* **6**, 27362; doi: 10.1038/srep27362 (2016).

## Supplementary Material

Supplementary Information
