# Supplementary material for: Divergence and Conservation of the Major UPR Branch IRE1-bZIP Signaling Pathway across Eukaryotes
Source: Sci Rep. 2016 Jun 3;6:27362. doi: 10.1038/srep27362 (PMC4891664; doi:10.1038/srep27362)
Supplement: Supplementary Information [file srep27362-s1.pdf]

Supplementary information

Divergence and Conservation of the Major UPR Branch IRE1-*bZIP Signaling* Pathway across Eukaryotes

Lingrui Zhang, Changwei Zhang, and Aiming Wang*

London Research and Development Centre, Agriculture and Agri-Food Canada, London, ON, N5V 4T3, Canada

Content

Supplementary Figures

Figure S1. DNA Sequences of *XBP1* Variants Used in this Study.

Figure S2. Subcellular Localization and Interactions of bZIP Orthologues.

a, The indicated YFP fusion proteins were transiently co-expressed in leaves of *N. benthamiana* along with the KDEL-mCherry ER marker and 3xNLS-CFP nucleus marker for 48 h, and fluorescent signals were monitored. N represents nucleus localization, and C cytosolic localization. Bars = 50 µm.

b, Transformed CRY1 *Δhac1*::TRP cells with CEN-ARS plasmid harboring *XBP1* U, *XBP1* S and *XBP1* Si, as described in Fig. 1, were sub-cultured in the absence or presence of 2% Gal for 8 h and subsequently treated without or with 10 mM DTT for 2 h, then RNA was extracted and RT-PCR was performed, as described previously[1], using primers given in Supplementary Table S1 and Fig. S1.

c, Detection of subcellular localization of XBP1 variants by confocal imaging of CRY1 *Δhac1*::TRP strains. The transformed yeast cells with 2µ plasmids were induced by galactose for 8 h and subjected to confocal observation. bZIP60 S was used as a positive control. Note that bZIP60 S and XBP1 S localized to the nuclei, whereas XBP1 U and XBP1 Si displayed YFP signal throughout the cytoplasm. Bars = 10 µm.

d, BiFC assay showed intra-species interactions, rather than inter-species interactions, among the spliced versions of bZIP60, HAC1p and XBP1 that were transiently co-expressed in leaves of *N. benthamiana* along with 3xNLS-RFP nucleus marker. BiFC signal was monitored at 2 dpi and was false-colored to appear green. Bars = 50 μm. Experiments were repeated three times with similar results.

Figure S3. The Interactions between ER-lumenal Domains of IRE1A and IRE1B *in Planta*.

a, Schematic of IRE1A and IRE1B. The ER-lumenal portion of IRE1A and IRE1B is divided in an N-terminal signal peptide domain (SP, green) and a core lumenal ER-stress-sensing domain (Sensor, yellow), which is tethered via a transmembrane domain (TMD, red) to IRE1A and IRE1B's cytosolic portion that is composed of a linker (light blue), a kinase (gray) and an RNase domain (purple). The number for the first amino acid of each domain and the number for the last amino acid of RNase domain were indicated. Note that the spl domain occurred in the main text consist of SP motif, sensor domain, TMD motif and linker domain, and IRE1A-S and IRE1B-S contains only the sensor domain from IRE1A and IRE1B, whereas sps domain contains both SP and sensor domains.

b, Homo-dimerization of IRE1A-sps and IRE1B-sps and hetero-interaction between IRE1A-SPS and IRE1B-SPS in leaves of *N. benthamiana* as determined by BiFC analysis. 3xNLS-CFP and KDEL-mCherry were used as nucleus and ER markers, respectively. The negative controls were performed on the indicated sets of constructs, showing no YFP signal. C marked right represents that the interaction takes place in cytosol. Bars = 25 μm.

c, Homo-dimerization of IRE1A-S and IRE1B-S and hetero-interaction between IRE1A-S and IRE1B-S were also determined by BiFC, as described in (**b**). C or N marked right the interaction in cytoplasm or nucleus, respectively. Bars = 25 μm.

d, Hetero-interaction between IRE1A and IRE1B was determined by BiFC, as described in (**b** and **c**). Bars = 25 μm. Experiments were repeated three times with similar results.

Figure S4. Molecular Phylogenetic Analysis of IRE1 Orthologues by Maximum Likelihood Method.

a, A unrooted phylogenetic history of 29 IRE1 orthologues was inferred by using the maximum likelihood method based on the Whelan and Goldman model[2]. The number on each branch represents confidence form 1,000 replication of bootstrap analysis. The tree is drawn to scale, with branch lengths measured in the number of substitutions per site. Note that 29 IRE1s belong to three groups and IRE1s tested in this study were highlighted in blue, and that of two human IRE1 homologues (IRE1α and IRE1β), IRE1α is well characterized and ubiquitously expressed, so we focused on it in this study.

b, The time-tree shown was generated using the RelTime method[2]. Divergence times for all branching points in the topology were calculated by using the maximum likelihood method. Branches colored in red

indicate that the IRE1p and IRE1B have a similar divergence time. Scale bar denotes the relative evolutionary time.

Figure S5. Conservation of the Core Lumenal Domains (Containing BiP Binding Motif) and the Cytosolic Domains of IRE1 Orthologues.

Bar diagrams depict the relative conservation of core lumenal domains (left panels) and cytosolic domains (right panels) of IRE1 homologues from fungi as well as orthologues from *C. reinhardtti*, plant and animal species. Conservation of residues among the indicated species was scored by using ClustalW on the platform of Jalview 2.9[3].

Figure S6. IRE1 Inhibitor Affects Root Meristem Size.

a, PCR with genomic DNA from the wild type (WT) and *ire1a-2 ire1b-4* was performed to test the homozygosis in the double mutant line by the indicated primer combinations. Primer sequences were given in Supplementary Table S1.

b, Root phenotype of the WT and *ire1a-2 ire1b-4* seedlings grown in the presence of 0.1% DMSO or 0.5 μM 4μ8C for 9 days.

c, Images of root meristems of the WT seedlings grown in the presence of 0.1% DMSO (control) or 0.5 μM 4μ8C. White arrowheads point to the quiescent center, and green arrowheads indicate the meristem transition zone. The cell wall (red) was stained with propidium iodide. Root meristem size, expressed as the number of cells in a cortex file from the quiescent center to the first elongated cortex cell (transition zone), was significantly reduced by the application of 4μ8C ($P < 0.001$), compared to control. Experiments were performed on 4-d-old seedlings. Bar = 100 μm.

Supplementary Table

Table S1. Primers Used in this Study.

Supplementary References

1 Zhang, L., Chen, H., Brandizzi, F., Verchot, J. & Wang, A. The UPR branch IRE1-*bZIP60* in plants plays an rssential role in viral infection and is complementary to the only UPR pathway in yeast. *PloS Genet* **11**, e1005164 (2015).

2 Tamura, K. *et al.* Estimating divergence times in large molecular phylogenies. *Proc Natl Acad Sci USA* **109**, 19333-19338 (2012).

3 Waterhouse, A. M., Procter, J. B., Martin, D. M., Clamp, M. & Barton, G. J. Jalview Version 2-a multiple sequence alignment editor and analysis workbench. *Bioinformatics* **25**, 1189-1191 (2009).

Figure S1. DNA Sequences of *XBP1* Variants Used in this Study.

XBP1 U sequence in pDONR221 (related to Fig. 1c, Fig. 2a-f and Fig. S2)

(Start codon colored in green, the first exon colored in grey, unconventional intron colored in purple, the second exon colored in blue, stop codon colored in red, primers used in Fig. S2 indicated in boxes)

ATGGTGGTGGTGGCAGCCGCGCCGAACCCGGCCGACGGACCCCTAAAGTTCTGCTTCTGTCGGGGCAGCCCGCCTCC
GCCGCCGGAGCCCCGGCCGGCCAGGCCCTGCCGCTCATGGTGCCAGCCCAGAGAGGGGCCAGCCCGGAGGCAGCGAG
CGGGGGGCTGCCCCAGGCGCGCAAGCGACAGCGCCTCACGCACCTGAGCCCCGAGGAGAAGGCGCTGAGGAGGAAA
CTGAAAAACAGAGTAGCAGCTCAGACTGCCAGAGATCGAAAGAAGGCTCGAATGAGTGAGCTGGAACAGCAAGTGGT
AGATTTAGAAGAAGAGAACCAAAAACTTTTGCTAGAAAATCAGCTTTTACGAGAGAAAACTCATGGCCTTGTAGTTGA
GAACCAGGAGTTAAGACAGCGCTTGGGGATGGATGCCCTGGTTGCTGAAGAGGAGGCGGAAGCCAAGGGGAATGAA
GTGAGGCCAGTGGCCGGGTCTGCTGAGTCCGCAGCACTCAGACTACGTGCACCTCTGCAGCAGGTGCAGGCCCAGTTG
TCACCCCTCCAGAACATCTCCCCATGGATTCTGGCGGTATTGACTCTTCAGATTCAGAGTCTGATATCCTGTTGGGCAT
TCTGGACAACTTGGACCCAGTCATGTTCTTCAAATGCCCTTCCCCAGAGCCTGCCAGCCTGGAGGAGCTCCCAGAGGTC
TACCCAGAAGGACCCAGTTCCTTACCAGCCTCCCTTTCTCTGTCAGTGGGGACGTCATCAGCCAAGCTGGAAGCCATTA
ATGAACTAA

XBP1 S sequence in pDONR221 (related to Fig. 1c, Fig. 2a-f and Fig. S2)

(Start codon colored in green, the first exon colored in grey, the elongated second exon colored in blue, the original stop codon in *XBP1* U shifted +2 thus underlined and colored in red, the newly introduced stop codon colored in red, primers used in Fig. S2 indicated in boxes)

ATGGTGGTGGTGGCAGCCGCGCCGAACCCGGCCGACGGACCCCTAAAGTTCTGCTTCTGTCGGGGCAGCCCGCCTCC
GCCGCCGGAGCCCCGGCCGGCCAGGCCCTGCCGCTCATGGTGCCAGCCCAGAGAGGGGCCAGCCCGGAGGCAGCGAG
CGGGGGGCTGCCCCAGGCGCGCAAGCGACAGCGCCTCACGCACCTGAGCCCCGAGGAGAAGGCGCTGAGGAGGAAA
CTGAAAAACAGAGTAGCAGCTCAGACTGCCAGAGATCGAAAGAAGGCTCGAATGAGTGAGCTGGAACAGCAAGTGGT
AGATTTAGAAGAAGAGAACCAAAAACTTTTGCTAGAAAATCAGCTTTTACGAGAGAAAACTCATGGCCTTGTAGTTGA
GAACCAGGAGTTAAGACAGCGCTTGGGGATGGATGCCCTGGTTGCTGAAGAGGAGGCGGAAGCCAAGGGGAATGAA
GTGAGGCCAGTGGCCGGGTCTGCTGAGTCCGCAGCAGGTGCAGGCCCAGTTGTCACCCCTCCAGAACATCTCCCCATG
GATTCTGGCGGTATTGACTCTTCAGATTCAGAGTCTGATATCCTGTTGGGCATTCTGGACAACTTGGACCCAGTCATGT
TCTTCAAATGCCCTTCCCCAGAGCCTGCCAGCCTGGAGGAGCTCCCAGAGGTCTACCCAGAAGGACCCAGTTCCTTAC
CAGCCTCCCTTTCTCTGTCAGTGGGGACGTCATCAGCCAAGCTGGAAGCCATTAATGAACTAATTCGTTTGACCACAT
ATATACCAAGCCCCTAGTCTTAGAGATACCCTCTGAGACAGAGAGCCAAGCTAATGTGGTAGTGAAAATCGAGGAAG
CACCTCTCAGCCCCTCAGAGAATGATCACCCTGAATTCATTGTCTCAGTGAAGGAAGAACCTGTAGAAGATGACCTCG
TTCCGGAGCTGGGTATCTCAAATCTGCTTTCATCCAGCCACTGCCCAAAGCCATCTTCCTGCCTACTGGATGCTTACAG
TGACTGTGGATACGGGGGTTCCCTTTCCCCATTCAGTGACATGTCCTCTCTGCTTGGTGTAAACCATTCTTGGGAGGAC
ACTTTTGCCAATGAACTCTTTCCCCAGCTGATTAGTGTCTAA

XBP1 Si sequence in pDONR221 (related to Fig. 1c and Fig. S2)

(*XBP1* Si represents that *XBP1* S contains the unconventional intron. Thus except the 26-nt of intron, all parts are the same as in the *XBP1* S. This construct produces XBP1 S by ending at the second stop codon underlined and colored in red if the unconventional editing takes place, otherwise produces XBP1 U by ending at the first stop codon colored in red, primers used in Fig. S2 indicated in boxes)

ATGGTGGTGGTGGCAGCCGCGCCGAACCCGGCCGACGGACCCCTAAAGTTCTGCTTCTGTCGGGGCAGCCCGCCTCC
GCCGCCGGAGCCCCGGCCGGCCAGGCCCTGCCGCTCATGGTGCCAGCCCAGAGAGGGGCCAGCCCGGAGGCAGCGAG
CGGGGGGCTGCCCCAGGCGCGCAAGCGACAGCGCCTCACGCACCTGAGCCCCGAGGAGAAGGCGCTGAGGAGGAAA
CTGAAAAACAGAGTAGCAGCTCAGACTGCCAGAGATCGAAAGAAGGCTCGAATGAGTGAGCTGGAACAGCAAGTGGT
AGATTTAGAAGAAGAGAACCAAAAACTTTTGCTAGAAAATCAGCTTTTACGAGAGAAAACTCATGGCCTTGTAGTTGA
GAACCAGGAGTTAAGACAGCGCTTGGGGATGGATGCCCTGGTTGCTGAAGAGGAGGCGGAAGCCAAGGGGAATGAA
GTGAGGCCAGTGGCCGGGTCTGCTGAGTCCGCAGCACTCAGACTACGTGCACCTCTGCAGCAGGTGCAGGCCCAGTTG
TCACCCCTCCAGAACATCTCCCCATGGATTCTGGCGGTATTGACTCTTCAGATTCAGAGTCTGATATCCTGTTGGGCAT
TCTGGACAACTTGGACCCAGTCATGTTCTTCAAATGCCCTTCCCCAGAGCCTGCCAGCCTGGAGGAGCTCCCAGAGGTC
TACCCAGAAGGACCCAGTTCCTTACCAGCCTCCCTTTCTCTGTCAGTGGGGACGTCATCAGCCAAGCTGGAAGCCATTA
ATGAACTAATTCGTTTGACCACATATATACCAAGCCCCTAGTCTTAGAGATACCCTCTGAGACAGAGAGCCAAGCTA
ATGTGGTAGTGAAAATCGAGGAAGCACCTCTCAGCCCCTCAGAGAATGATCACCCTGAATTCATTGTCTCAGTGAAGG
AAGAACCTGTAGAAGATGACCTCGTTCCGGAGCTGGGTATCTCAAATCTGCTTTCATCCAGCCACTGCCCAAAGCCAT
CTTCCTGCCTACTGGATGCTTACAGTGACTGTGGATACGGGGGTTCCCTTTCCCCATTCAGTGACATGTCCTCTCTGCTT
GGTGTAAACCATTCTTGGGAGGACACTTTTGCCAATGAACTCTTTCCCCAGCTGATTAGTGTCTAA

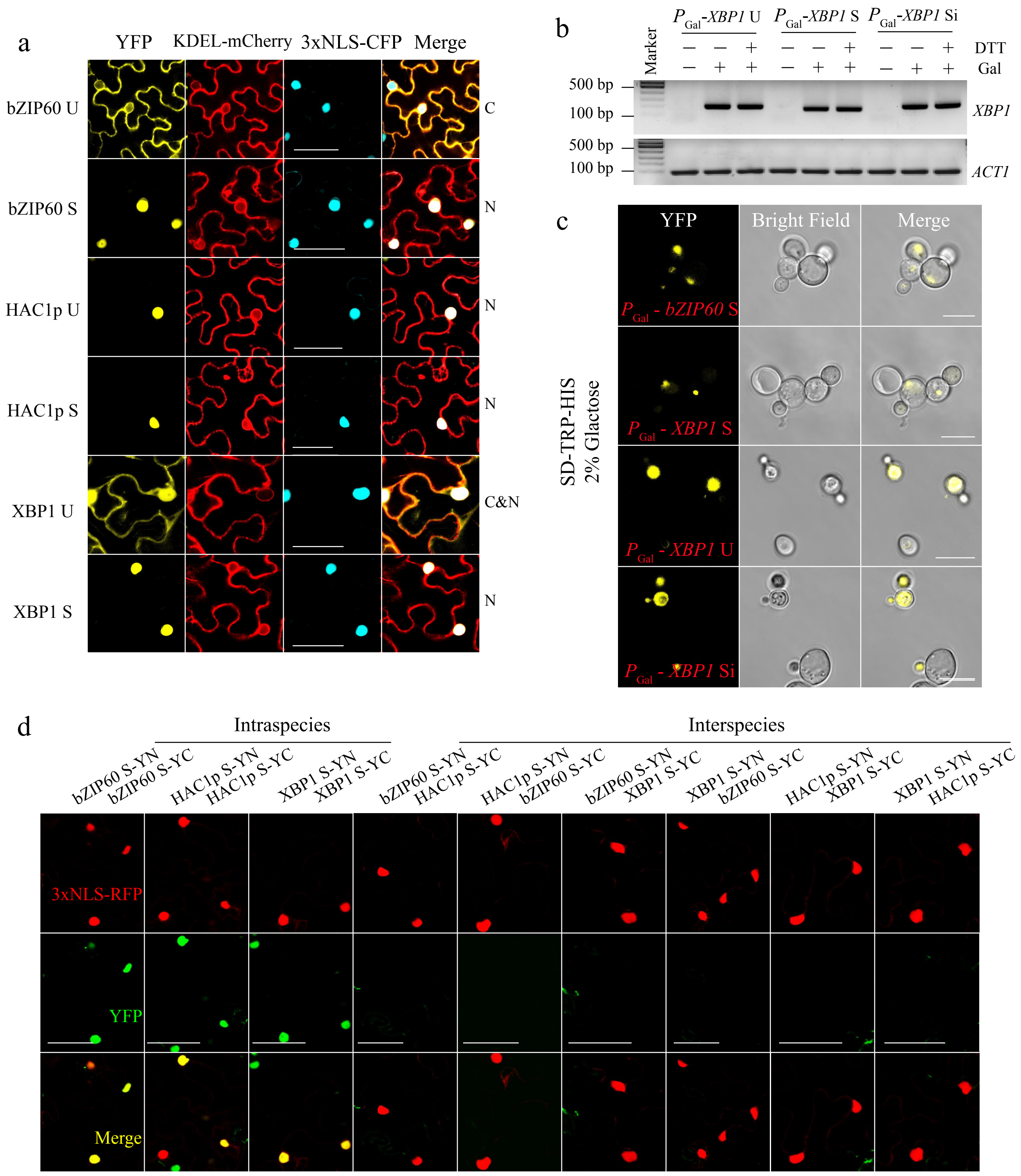

Figure S2. Subcellular Localization and Interactions of bZIP Orthologues.

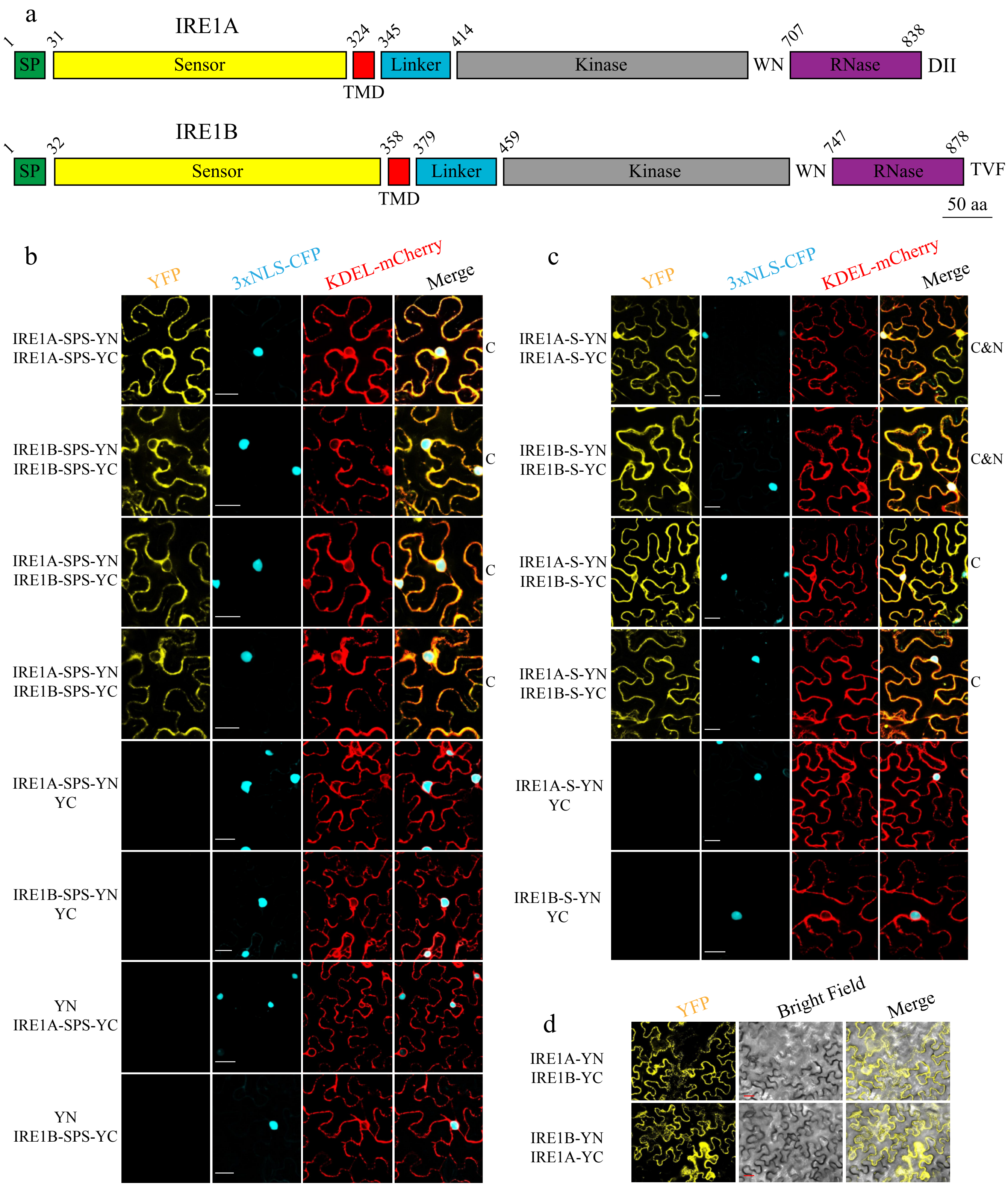

Figure S3. The Interactions between ER-lumenal Domains of IRE1A and IRE1B in Planta.

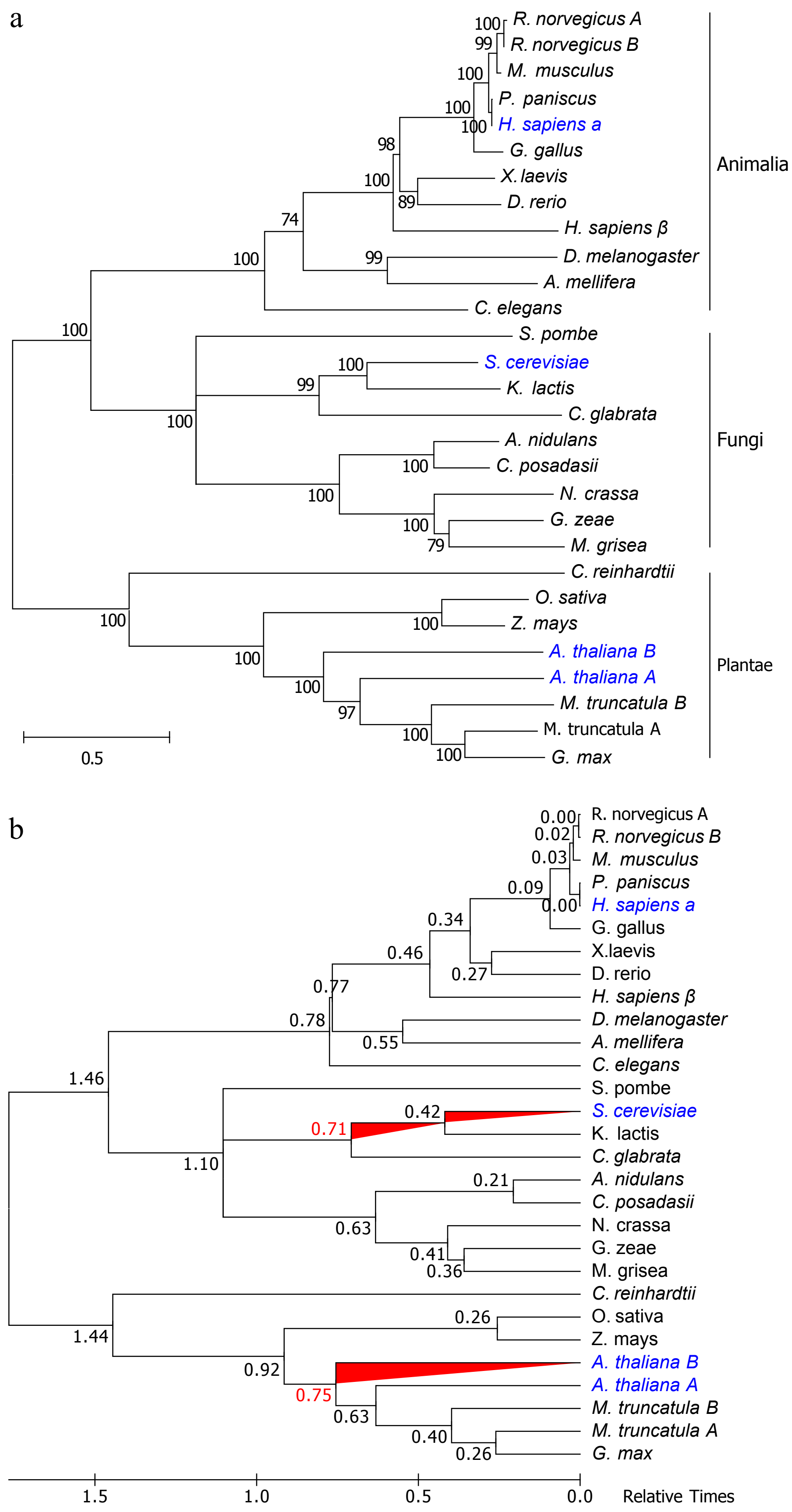

Figure S4. Molecular Phylogenetic Analysis of IRE1 Orthologues by Maximum Likelihood Method.

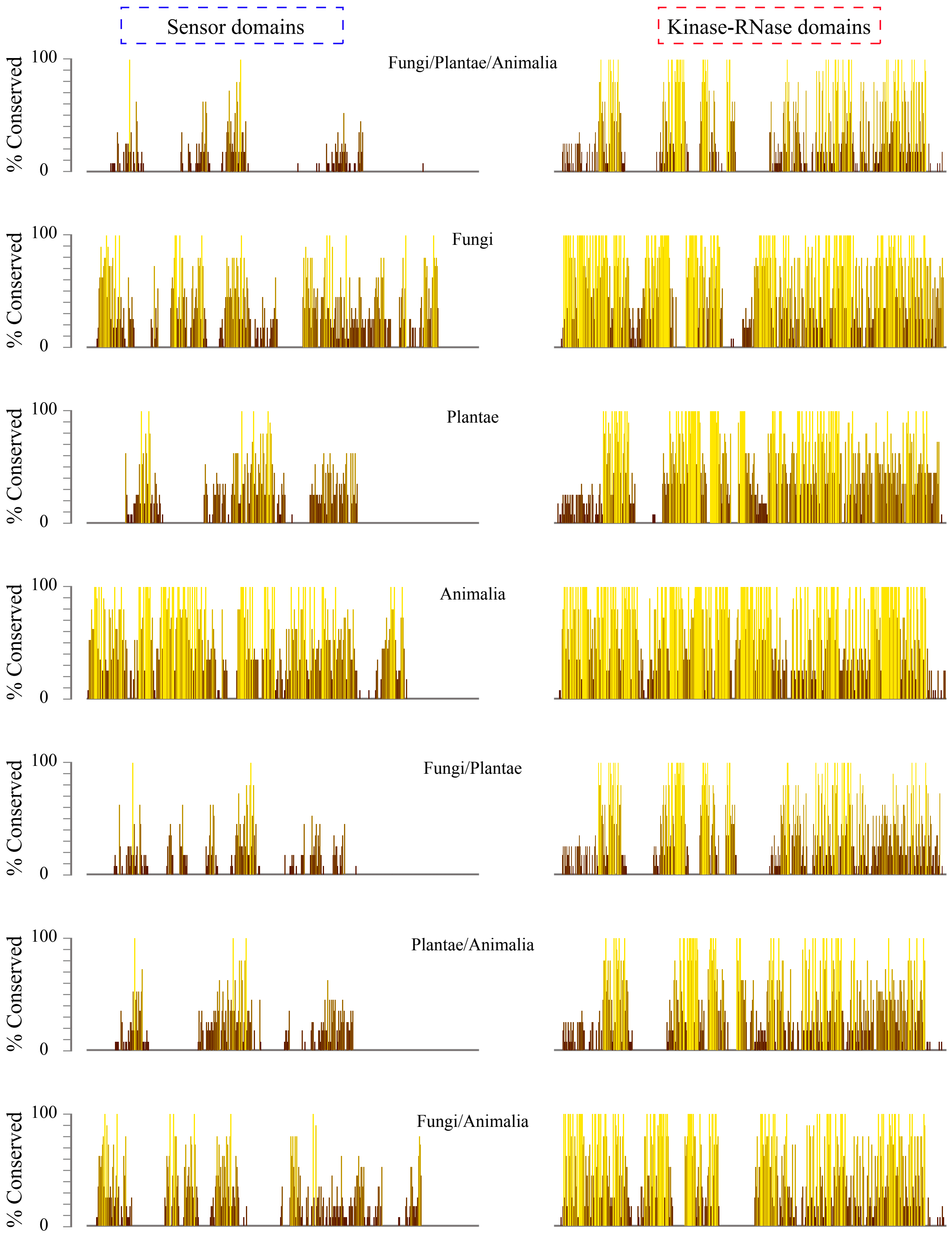

Figure S5. Conservation of the Core Lumenal Domains (Containing BiP Binding Motif) and the Cytosolic Domains of IRE1 Orthologues.

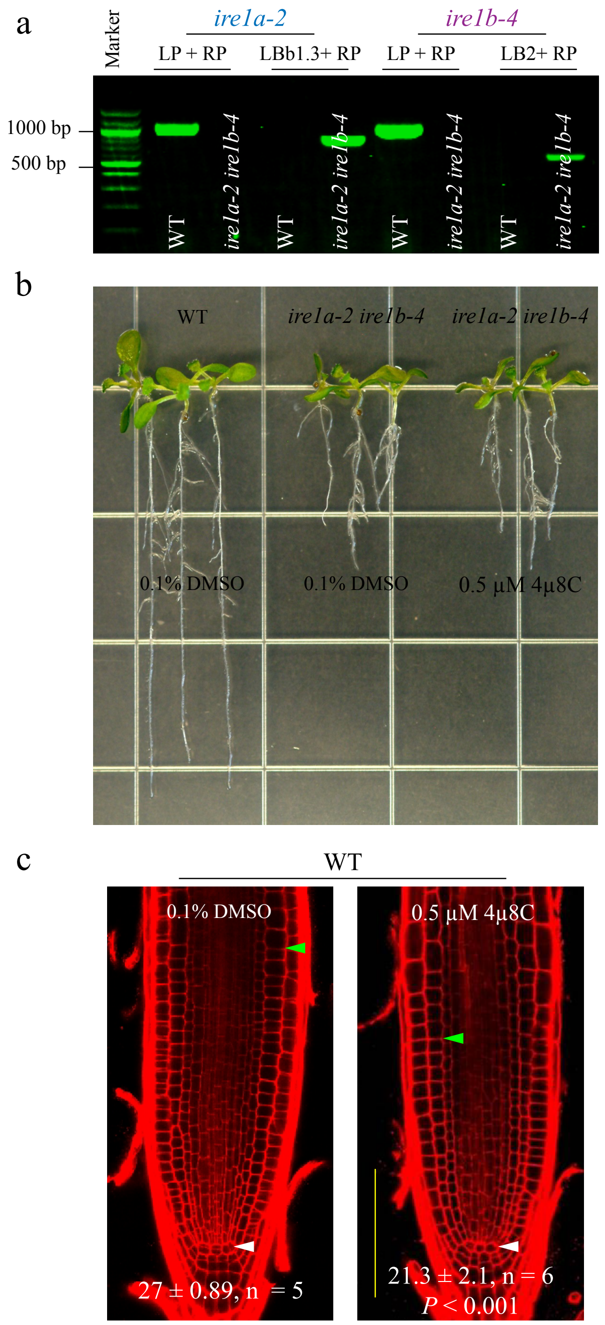

Figure S6. IRE1 Inhibitor Affects Root Meristem Size.

Table S1. Primers Used in this Study.

Name	Sequences (5’-3’)	Purpose/Note	Related Figures
LBb1.3	ATTTTGCCGATTTCGGAAC	Genotyping analysis of *Arabidopsis*<br><br>Note:<br>LBb1.3 for SALK lines<br>LB2 for SAIL lines	Related to Fig. S6
LB2	GCTTCCTATTATATCTTCCCAAATTACCAATACA		
ire1a-2 LP	TGCGTTCAGACACTAACATGC		
ire1a-2 RP	GAAGAAGAACCGTAAATCCGC		
ire1b-4 LP	CCTCTCGAACCCTTCAGGTAC		
ire1b-4 RP	GAAGGAAAACGGACATCCTTC		
sY2HhIRE1 F	cgcagagtggccattacggccATGCCGGCCCGGCGGCTGCTGC	Constructing IRE1 orthologues split-ubiquitin Y2H vectors<br><br>Note:<br>The underlined nucleotides indicate *Sfi* I sites.	Related to Fig. 3f, g
sY2HhIRE1 R	tctcgagaggccgaggcggccttGAGGGCGTCTGGAGTCACTGGGGGCT		
sY2HIRE1B F	cgcagagtggccattacggccATGAGAGGATCTGCACTACTT		
sY2HIRE1B R	tctcgagaggccgaggcggccttGAATACAGTGGTCTTAGAGTA		
sY2HIRE1A F	cgcagagtggccattacggccATGCCGCCGAGATGTCCTTTC		
sY2HIRE1A R	tctcgagaggccgaggcggccttGATGATGTCGCATTTGAAGTA		
sY2HyIRE1p F	cgcagagtggccattacggccATGCGTCTACTTCGAAGAAACATGT		
sY2HyIRE1p R	tctcgagaggccgaggcggccttTGAATACAAAAATTCACGTAAAATTTG		
dIRE1A F	ggggacaagtttgtacaaaaaagcaggcttcATGCCGCCGAGATGTCCTTTCC	Constructing IRE1A and IRE1B’s spl, SP-sensor and sensor domains<br><br>Note:<br>The sequences in lowercase represent the *aat*B Gateway arms	Related to Fig. 3d, e and Fig. S3
dIRE1A spl R	ggggaccactttgtacaagaaagctgggtcCAACTTACCGATCTTTCTCCCATCAG		
dIRE1A S F	ggggacaagtttgtacaaaaaagcaggcttcATGGACGATGTGACGTATCCGATCG		
dIRE1A S R	ggggaccactttgtacaagaaagctgggtcTTGTCCAAACAAATATGTATATTTCTG CTTATG		
dIRE1B F	ggggacaagtttgtacaaaaaagcaggcttcATGAGAGGATCTGCACTACTTGA		
dIRE1B spl R	ggggaccactttgtacaagaaagctgggtcCAGTTTACCAACCCTATATCCTTC		
dIRE1B S F	ggggacaagtttgtacaaaaaagcaggcttcATGTTCAAAGGATCTGAAATCTCCAA G		
dIRE1B S R	ggggaccactttgtacaagaaagctgggtcAGAAAATTTGCTAGCAAAGCCTGC		
dIRE1p F	ggggacaagtttgtacaaaaaagcaggcttcATGCGTCTACTTCGAAGAAACATGT	Constructing IRE1p and hIRE1’s spl domains<br><br>Note:<br>The sequences in lowercase represent the *aat*B Gateway arms	Related to Fig. 3d, e
dIRE1p spl R	ggggaccactttgtacaagaaagctgggtcCAAATTTTTTAAAGATTGCTCAAAGTT		
dhIRE1 F	ggggacaagtttgtacaaaaaagcaggcttcATGCCGGCCCGGCGGCTGCTGCTGC		
dhIRE1 spl R	ggggaccactttgtacaagaaagctgggtcGGAAATTTTCCCAACTATCAC		
3xNLS(atg) F	ggggacaagtttgtacaaaaaagcaggcttcATGATGCAGCCTAAGAAGAAGAGAA AGGTTGGAGGACAACCGAAAAAGAAAAGGAAAGTCGGCGGTCAG CCCAAGAAAAAGCGGAAGGGTGGG	Creating 35S::3xNLS-CYF and 35S::3xNLS-RYF reporters<br><br>Note:<br>The sequences in lowercase represent the *aat*B Gateway arms	Related to Fig. 2f, Fig. S2 and Fig. S3
3xNLS(atg) R	ggggaccactttgtacaagaaagctgggtcCCCACCCTTCCGCTTTTTCTTGGGCTGA CCGCCGAC		
ACT1-F	GACTGAAGCTCCAATGAAC	RT-PCR analysis in yeast	Related to Fig. S2
ACT1-R	GGCTTGGATGGAAACGTAGA		
